# Increased trabecular bone and improved biomechanics in an osteocalcin-null rat model created by CRISPR/Cas9 technology

**DOI:** 10.1242/dmm.025247

**Published:** 2016-10-01

**Authors:** Laura J. Lambert, Anil K. Challa, Aidi Niu, Lihua Zhou, Janusz Tucholski, Maria S. Johnson, Tim R. Nagy, Alan W. Eberhardt, Patrick N. Estep, Robert A. Kesterson, Jayleen M. Grams

**Affiliations:** 1 Department of Genetics, University of Alabama at Birmingham, Birmingham, AL 35294, USA; 2 Department of Surgery, University of Alabama at Birmingham, Birmingham, AL 35294, USA; 3 Department of Nutrition Sciences, University of Alabama at Birmingham, Birmingham, AL 35294, USA; 4 Department of Biomedical Engineering, University of Alabama at Birmingham, Birmingham, AL 35294, USA; 5 Department of Surgery, Birmingham VA Medical Center, Birmingham, AL 35233, USA

**Keywords:** Osteocalcin, Bone strength, Bone structure, Genetic animal models, Osteocalcin knockout

## Abstract

Osteocalcin, also known as bone γ-carboxyglutamate protein (Bglap), is expressed by osteoblasts and is commonly used as a clinical marker of bone turnover. A mouse model of osteocalcin deficiency has implicated osteocalcin as a mediator of changes to the skeleton, endocrine system, reproductive organs and central nervous system. However, differences between mouse and human osteocalcin at both the genome and protein levels have challenged the validity of extrapolating findings from the osteocalcin-deficient mouse model to human disease. The rat osteocalcin (*Bglap*) gene locus shares greater synteny with that of humans. To further examine the role of osteocalcin in disease, we created a rat model with complete loss of osteocalcin using the CRISPR/Cas9 system. Rat osteocalcin was modified by injection of CRISPR/Cas9 mRNA into the pronuclei of fertilized single cell Sprague-Dawley embryos, and animals were bred to homozygosity and compound heterozygosity for the mutant alleles. Dual-energy X-ray absorptiometry (DXA), glucose tolerance testing (GTT), insulin tolerance testing (ITT), microcomputed tomography (µCT), and a three-point break biomechanical assay were performed on the excised femurs at 5 months of age. Complete loss of osteocalcin resulted in bones with significantly increased trabecular thickness, density and volume. Cortical bone volume and density were not increased in null animals. The bones had improved functional quality as evidenced by an increase in failure load during the biomechanical stress assay. Differences in glucose homeostasis were observed between groups, but there were no differences in body weight or composition. This rat model of complete loss of osteocalcin provides a platform for further understanding the role of osteocalcin in disease, and it is a novel model of increased bone formation with potential utility in osteoporosis and osteoarthritis research.

## Abstract (precis)

**Summary:** A complete null of osteocalcin, generated by the CRISPR/Cas9 system, results in an increase in trabecular bone, increased bone strength and altered glucose homeostasis in Sprague-Dawley rats.

## INTRODUCTION

Osteocalcin, also known as bone γ-carboxyglutamate protein (Bglap), is the most abundant noncollagenous protein in bone and comprises ∼1% of total body protein (McGuigan et al., 2010). An osteocalcin-deficient mouse model displays increased cortical bone thickness and density, trabecular bone and bone strength (Ducy et al., 1996). This osteocalcin-deficient mouse model generated much interest outside of bone formation due to additional metabolic, reproductive and neurological phenotypes (Lee et al., 2007; Oury et al., 2011, 2013; Karsenty and Oury, 2012). The mice displayed obesity, and decreased insulin sensitivity and glucose tolerance (Lee et al., 2007). Osteocalcin was later shown to act as a regulator of male fertility, resulting in smaller reproductive organs and lower circulating testosterone (Oury et al., 2011); and to influence cognition by modulating neurotransmitter synthesis (Oury et al., 2013).

The contribution of osteocalcin to bone structure and function, metabolism, male fertility and cognition in humans remains to be determined (Li et al., 2016). There are considerable differences between the mouse and human osteocalcin gene loci, complicating interpretation of the results from the osteocalcin-deficient mouse model and raising the possibility that data from the mouse model might not be pertinent to human disease (Booth et al., 2013, 2014). For example, the mouse osteocalcin gene locus underwent a triplication event resulting in two functional copies of osteocalcin expressed in bone (*Bglap-1* and *Bglap-2*) and an additional copy expressed in other non-osteoid tissues (*Bglap-3*) (Desbois et al., 1994). In the osteocalcin-deficient mouse model, the entire *Bglap-2* sequence to exon 4 of *Bglap-1* was deleted (Ducy et al., 1996). The *Bglap-3* gene remained intact. Further confounding interpretation of data from the mouse model is the interspersal of a progestin and adiponectin receptor, *Paqr6*, that also underwent triplication in the gene locus. Two putative *Paqr6* genes were included in the deletion to create the osteocalcin-deficient animal. Similar to humans, the rat osteocalcin gene locus consists of a single copy of osteocalcin. Like the human osteocalcin gene, transcription of the rat osteocalcin gene is upregulated by vitamin D, whereas the mouse osteocalcin genes are downregulated by vitamin D (Kesterson et al., 1993; Arbour et al., 1995; Javed et al., 1999; Booth et al., 2013). This similarity and synteny between rat and human osteocalcin gene loci suggests that the rat might be a more appropriate animal model system to investigate osteocalcin function, particularly as it pertains to relevance in human disease. In this paper, we address this issue and report the generation and initial characterization of an osteocalcin-null mutant rat model.

## RESULTS

### Generation of a osteocalcin-null rat using CRISPR/Cas9 system

**Fig. 1.**
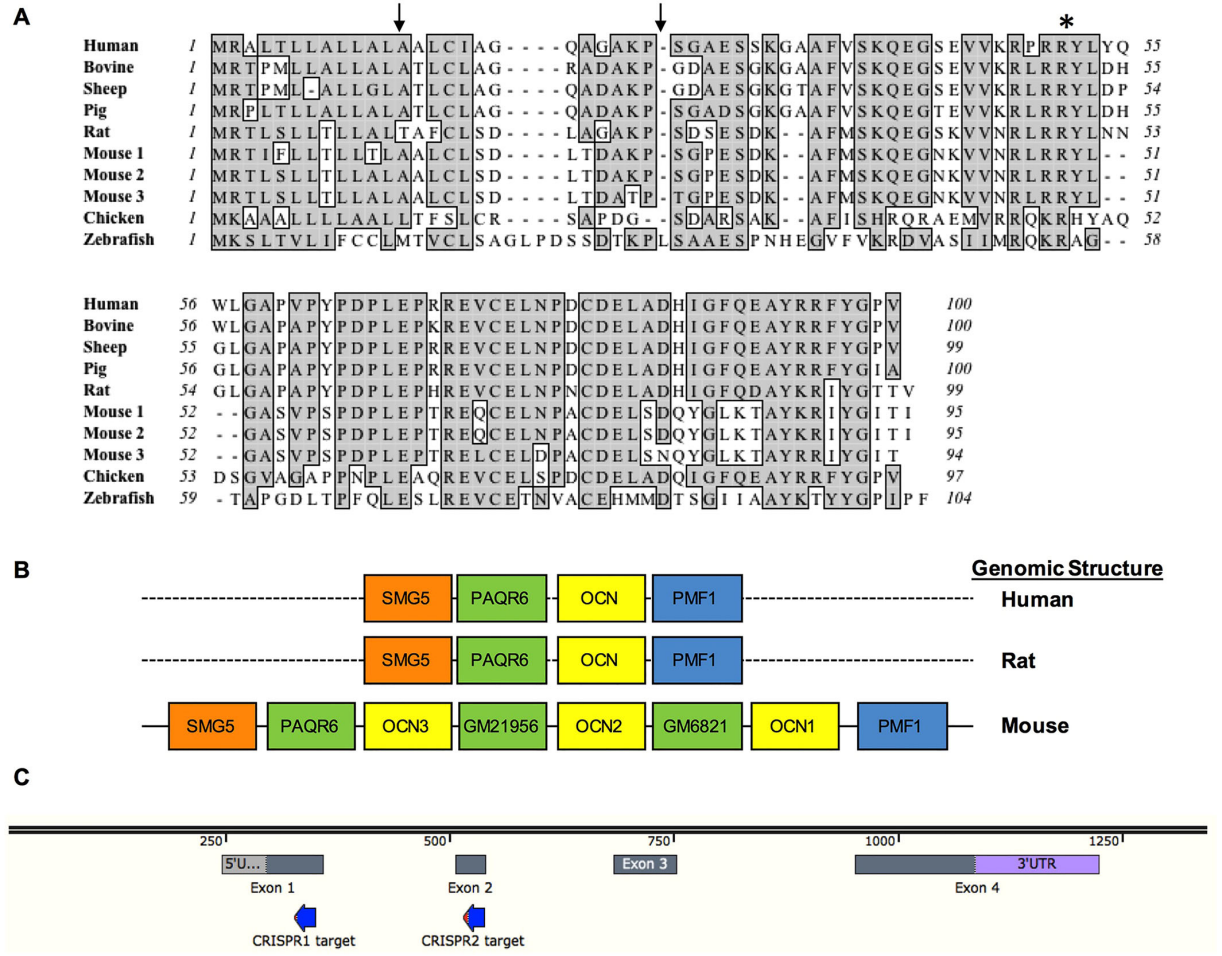
**Comparison of the** **similarity** **and synteny of osteocalcin between species and CRISPR guide design.** (A) Peptide sequence of different species. Gray shading indicates conserved amino acid residues. The asterisk indicates a cleavage site between a prepropeptide and mature protein regions. CRISPR cut sites are indicated by arrows above the sequence. (B) Structure of the genomic locus of human and rat versus mouse osteocalcin. A triplication of osteocalcin and the interspersed progestin and adiponectin receptor 6 (*Paqr6*) gene occurred in the mouse but not in the human or rat osteocalcin gene locus. (C) Design of the two CRISPR guide RNAs to exons 1 and 2 of the rat gene.

The high degree of similarity and synteny between the rat and human osteocalcin gene loci (Fig. 1A,B) indicated that targeting the osteocalcin gene early in the protein sequence would create a model analogous to loss of osteocalcin in humans. Thus, two CRISPR guide RNAs were designed to target exons 1 and 2 of osteocalcin to disrupt the protein early in the amino acid sequence (Fig. 1C). The CRISPR guide RNA and Cas9 mRNA mixtures were microinjected into the pronuclei of 33 fertilized embryos of Sprague-Dawley rats and transferred to pseudopregnant female rats, of which 21 pups were born (64%).

### Multiple alleles result in complete loss of osteocalcin protein

**Fig. 2.**
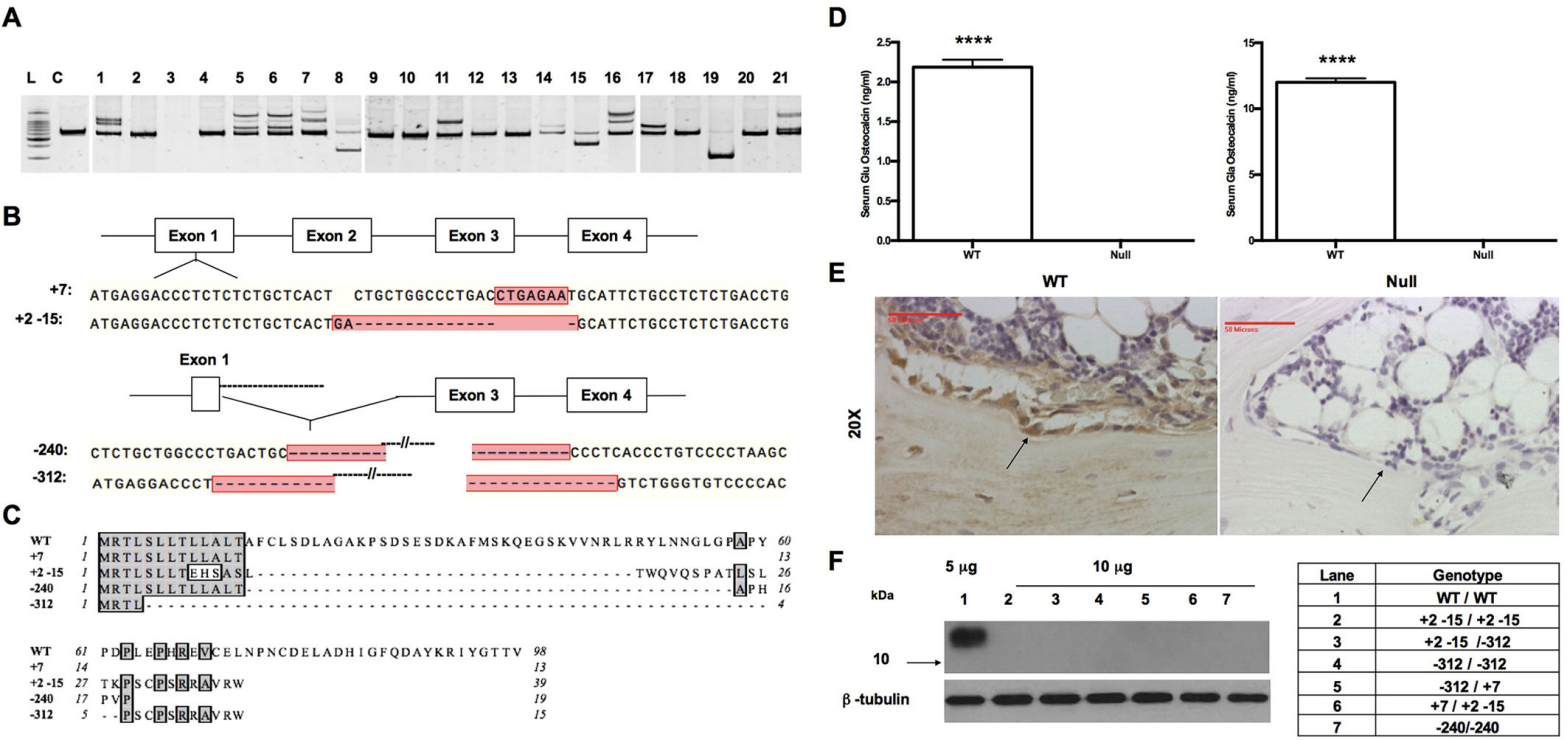
**Identification of founder animals and multiple null alleles with indels.** (A) Heteroduplex mobility assay of genomic DNA from F0 animals born from microinjection with CRISPR/Cas9. Founder 1 was mated to founder 19 to establish the osteocalcin colony. L, ladder; C, wild-type control. (B) Mutant alleles +7, +2 –15, –240 and –312 transmitted to the F1 generation are indicative of mosaicism in the founder germlines. (C) Predicted translations of mutant alleles. (D) Serum Glu-osteocalcin, and serum Glu- and Gla-osteocalcin levels for wild-type (WT) and osteocalcin-null rats. Results are mean±s.e.m. *n*=3 for WT and null. ****P<0.0001 (two-tailed parametric unpaired *t*-test). (E) Osteocalcin immunohistochemistry. Black arrows indicate positive (WT, left) and negative (null, right) osteoblasts. (F) Western blot of femurs from compound heterozygous F2 animals demonstrate complete loss of osteocalcin protein.

Genotyping was performed by amplifying a 601-bp fragment encompassing the CRISPR single guide RNA (sgRNA) target sites from tail genomic DNA samples, followed by a heteroduplex mobility assay (HMA). Based on HMA profiles, the presence of indels was identified in 12 of 21 (58%) pups (Fig. 2A) born from the first set of microinjected embryos. The alleles were confirmed by Sanger sequencing, which revealed frameshift mutations. Multiple alleles were present in many founder animals and indicated mosaicism (Fig. 2B). Founders 1 and 19 were mated to each other in order to establish germline transmission of mutant alleles including a 7-bp insertion (+7), a compound 2-bp insertion and a 15-bp deletion (+2–15), and a 312-bp deletion (–312). A second set of pups born from the CRISPR microinjection yielded an additional founder animal that transmitted a 240-bp deletion (–240, data not shown). All of these alleles were predicted to cause frameshift mutations resulting in premature stop codons (Fig. 2C). Total loss of osteocalcin protein was demonstrated by complete loss of both γ-carboxylated (Gla-) and uncarboxylated (Glu-) forms of osteocalcin in the serum of the osteocalcin-null male rats as determined by enzyme-linked immunosorbent assay (ELISA) (Fig. 2D). Western blots of tissue samples (brain, heart, lung, bone, gonadal fat, subcutaneous fat, quadriceps muscle, liver, kidney, pancreas and spleen) from wild-type animals demonstrated that osteocalcin was only expressed in bone (data not shown); there was no osteocalcin detectable in any of these tissues taken from a homozygous 312-bp deletion (–312) F2 generation animal as expected (data not shown). Loss of osteocalcin protein in the null mutant animals was further confirmed by immunohistochemistry on sectioned femurs (Fig. 2E) and by western blot analyses of protein isolated from whole tibiae of animals with compound heterozygous combinations of these alleles (Fig. 2F), in contrast to the robust signal from wild-type animals.

### Loss of osteocalcin does not affect body composition

**Fig. 3.**
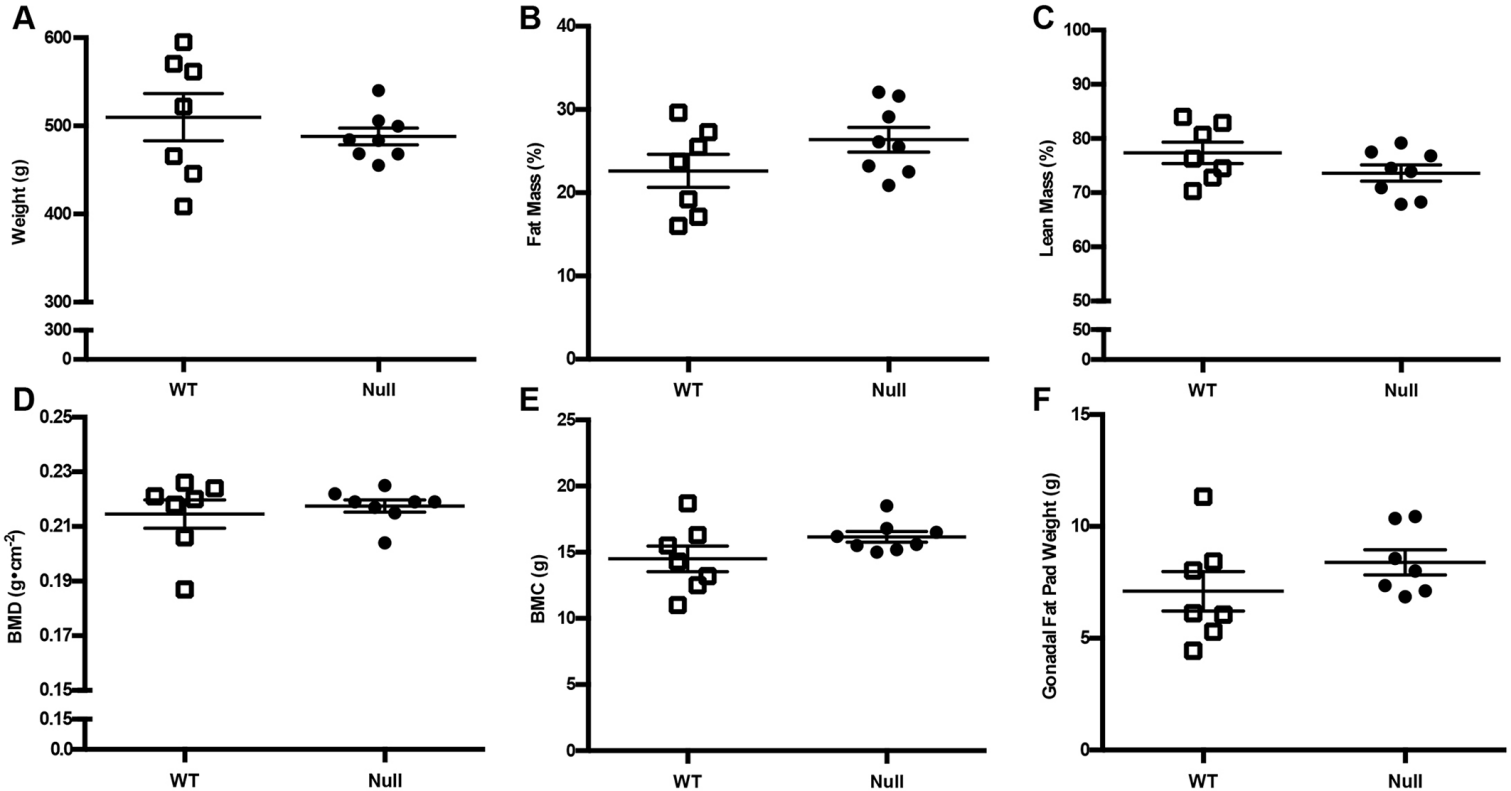
**DXA analysis of wild-type and osteocalcin-null male rats.** Wild-type (WT) and osteocalcin-null male rats were analyzed for (A) weight, *P*=0.4339, (B) percentage fat mass, *P*=0.1490, (C) percentage lean mass, *P*=0.1517, (D) bone mineral density, *P*=0.5958, (E) bone mineral content, *P*=0.1215, and (F) gonadal fat pad weight, *P*=0.2434. *n*=7 WT, *n*=8 null. Results are mean±s.e.m. *P*-values were calculated with a two-tailed parametric unpaired *t*-test.

5-month-old wild-type and osteocalcin-null male rats were scanned by dual-energy X-ray absorptiometry (DXA) for total body composition. Total body weight, the percentage fat mass, percentage lean mass, bone mineral density (BMD) and bone mineral content (BMC) were not significantly different (Fig. 3A–E). Gonadal fat pads were excised from the males of each cohort and weighed. There was no significant difference between the two groups (Fig. 3F).

### Loss of osteocalcin results in differences in glucose and insulin tolerance

**Fig. 4.**
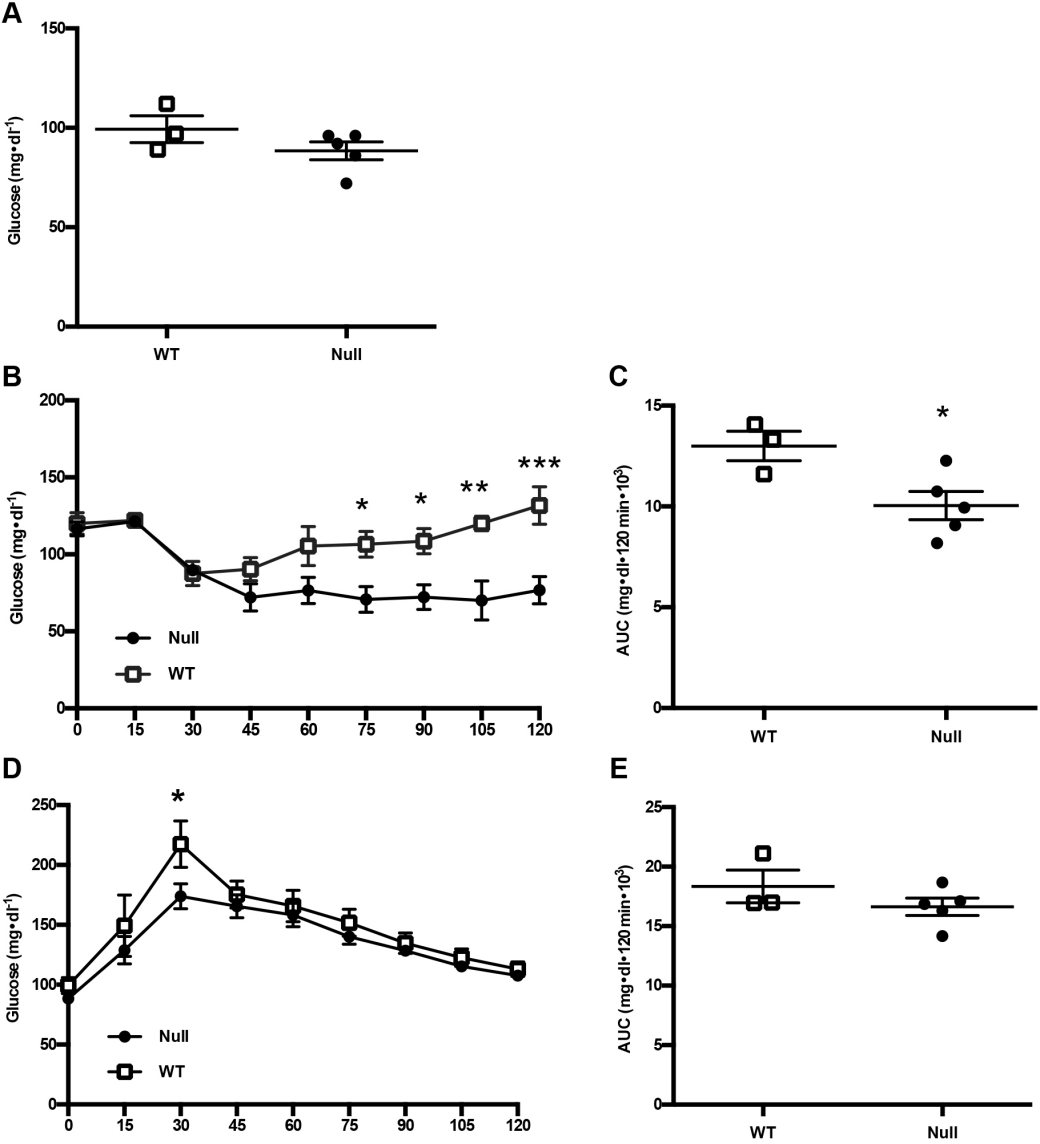
**Glucose and insulin tolerance tests in wild-type and osteocalcin-null male rats.** Wild-type (WT) and osteocalcin-null male rats were analyzed for (A) fasting glucose levels, *P*=0.2080, and by (B) an insulin tolerance test and (D) a glucose tolerance test. (C) Quantification of insulin tolerance test results, *P*=0.0337. (E) Quantification of glucose tolerance test results, *P*=0.2656. *n*=3 WT, *n*=5 null. Results are mean±s.e.m. *P≤0.05, **P≤0.01, ***P≤0.001. *P*-values were calculated with a two-tailed parametric unpaired *t*-test (C,E) and two-way ANOVA (B,D).

Fasting glucose measurements were assessed from fresh tail blood in 5-month-old male rats of osteocalcin-null and wild-type rats immediately prior to challenge with glucose or insulin for glucose and insulin tolerance tests, respectively. There was no significant difference between groups for the fasting glucose level (Fig. 4A; *P*=0.2080); however, at 75 min after insulin injection the groups began to diverge (Fig. 4B,C), indicating an increase in insulin sensitivity in osteocalcin-null animals (10.04±0.70 mg dl^−1^ and 13.00±0.73 mg dl^−1^ for null versus WT animals, respectively; mean±s.e.m., *P*=0.0337). Consistent with an increase in insulin sensitivity, osteocalcin-null animals exhibited significantly lower blood glucose levels at 30 min post glucose injection (Fig. 4D,E; 16.62±0.73 mg dl^−1^ versus 18.34±1.38 mg dl^−1^, respectively; *P*=0.0279).

### Loss of osteocalcin affects trabecular bone measurements

**Fig. 5.**
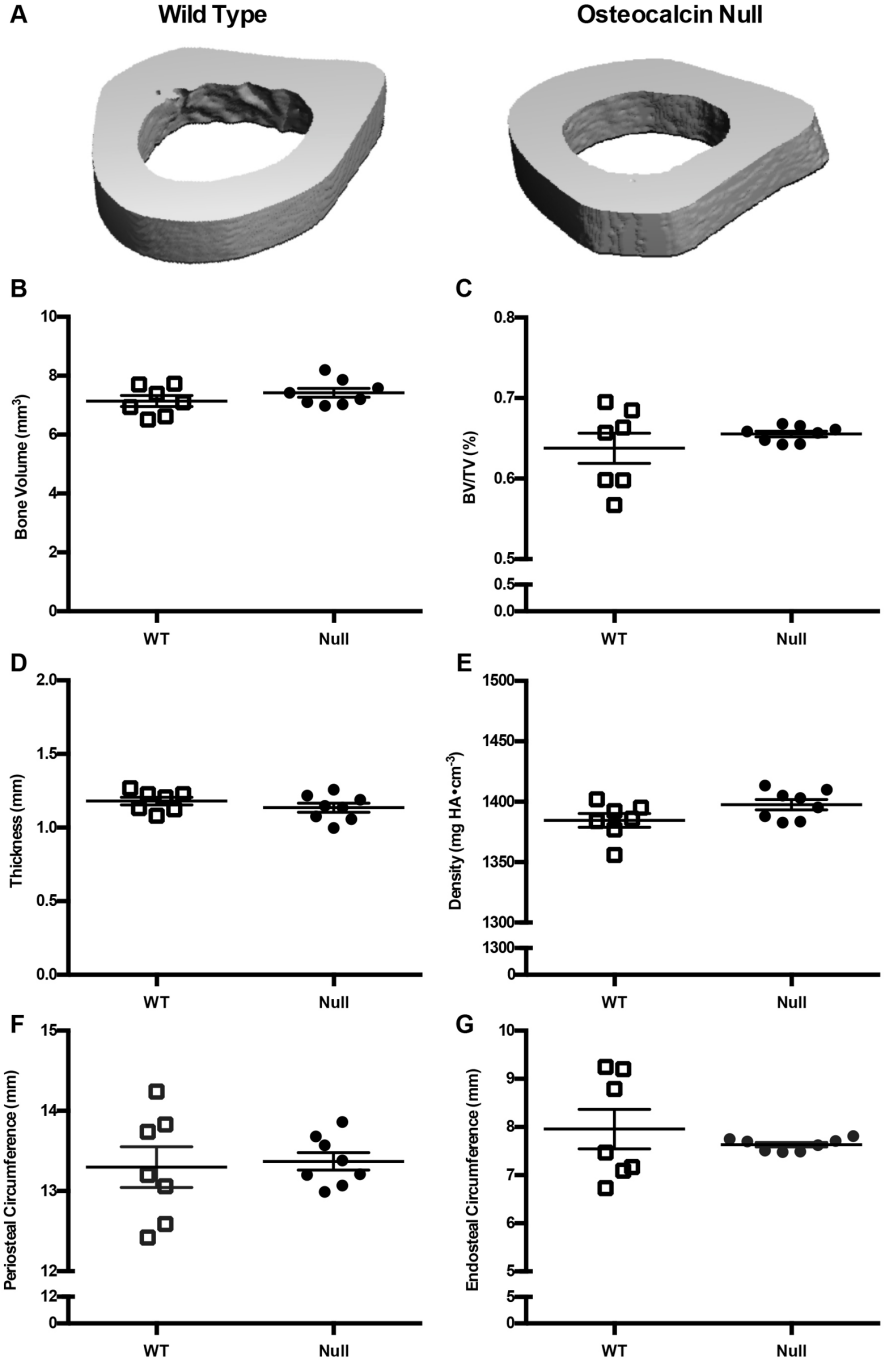
**Cortical µCT from wild-type and osteocalcin-null male rats.** (A) Representative µCT image of rat femoral cortical bone from a wild-type (WT, left) and an osteocalcin-null (right) rat. (B–G) Quantification of µCT data. (B) Cortical bone volume, *P*=0.2585, (C) cortical bone volume/tissue volume, *P*=0.3378, (D) cortical thickness, *P*=0.2876, (E) cortical density, *P*=0.0845. *n*=8 WT, *n*=8 null. (F) Periosteal circumference, *P*=0.7865. (G) Endosteal circumference, *P*=0.4144. *n*=7 WT, *n*=8 null. Results are mean±s.e.m. *P*-values were calculated with a two-tailed parametric unpaired *t*-test.

**Fig. 6.**
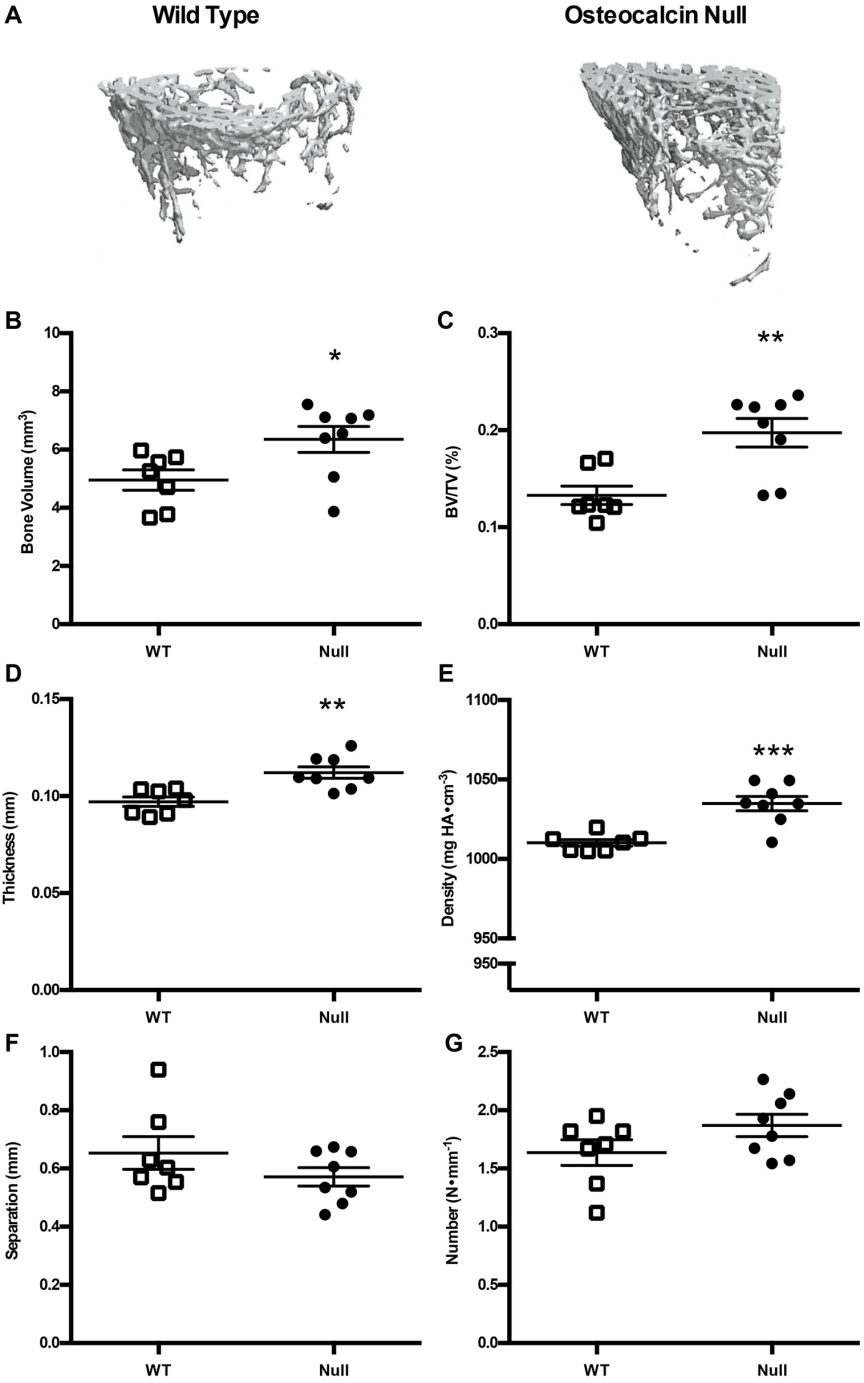
**Trabecular µCT from wild-type and osteocalcin-null male rats.** (A) Representative µCT image of rat femoral trabecular bone from a wild-type (WT, left) and an osteocalcin-null (right) rat. (B–G) Quantification of µCT data. (B) Trabecular bone volume, *P*=0.0316, (C) trabecular bone volume/tissue volume, *P*=0.0035, (D) trabecular thickness, *P*=0.0021, (E) trabecular density, *P*=0.0004, (F) trabecular separation, *P*=0.2130 and (G) trabecular number, *P*=0.1332. *n*=7 WT, *n*=8 null. Results are mean±s.e.m. *P*-values were calculated with a two-tailed parametric unpaired *t*-test. *P≤0.05, **P≤0.01, ***P≤0.001.

Osteocalcin-null and wild-type control animals were sacrificed at 5 months of age and the femurs excised for further analysis by microcomputed tomography (μCT). Cortical bone volume (*P*=0.2585), percentage bone volume (*P*=0.3378), thickness (*P*=0.2876), density (*P*=0.0845), and average periosteal circumference (*P*=0.7865) and endosteal circumference (*P*=0.4144) were not significantly different between groups (Fig. 5A–G). For trabecular bone, there was an increase in bone volume in osteocalcin-null rats compared to control animals (4.96±0.35 mm^3^ and 6.35±0.44 mm^3^, respectively; *P*=0.0316) (Fig. 6B). Trabecular bone measurements in null animals also differed significantly from wild-type in percentage bone volume (*P*=0.0035), thickness (*P*=0.0021) and density (*P*=0.0004) (Fig. 6C–E). Trabecular separation (*P*=0.2130) and number (*P*=0.1332) were not different between groups (Fig. 6F,G).

### Loss of osteocalcin affects femoral biomechanics

**Fig. 7.**
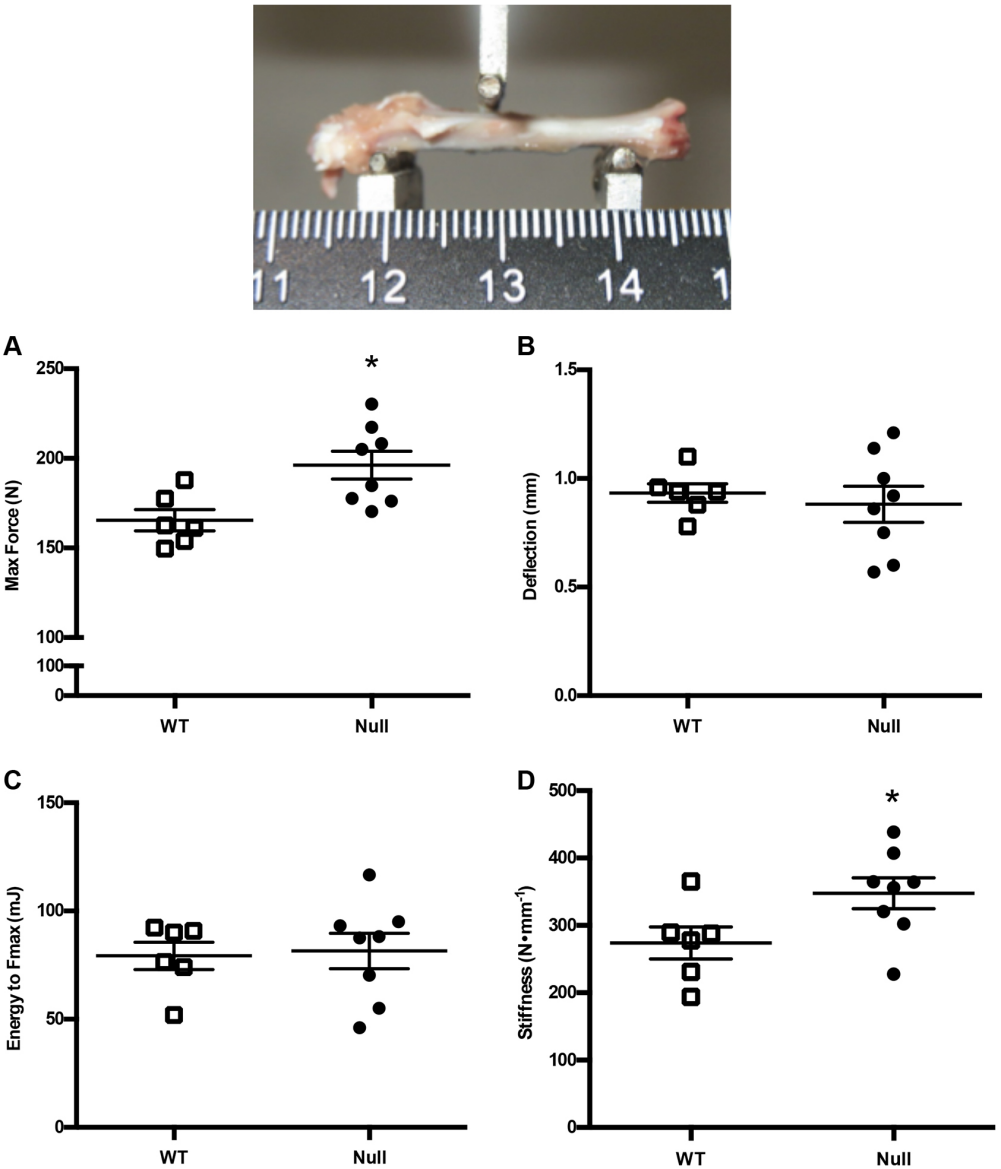
**Three-point break biomechanical assay.** Wild-type (WT) and osteocalcin-null male rats were analyzed for (A) maximum force, *P*=0.0118, (B) deflection, *P*=0.6233, (C) Energy to *F*_max_, *P*=0.8405, (D) stiffness, *P*=0.0492. *n*=6 WT, *n*=8 null. Results are mean±s.e.m. *P*-values were calculated with a two-tailed parametric unpaired *t*-test. *P≤0.05.

Excised femurs from 5-month-old male wild-type and osteocalcin-null rats were subjected to a three-point break assay to determine the strength and stiffness of the bone. This assay revealed that there was a significant increase in the maximum force needed to break the femurs of osteocalcin-null rats compared to wild-type animals (196.2±7.62 N and 165.5±5.93 N, respectively; mean±s.e.m., *P*=0.0118) (Fig. 7A). The osteocalcin-null animals also had a significant increase in stiffness (347.6±22.97 N mm^−1^ versus 274±23.78 N mm^−1^; *P*=0.0492) (Fig. 7D). There was no difference in deflection or energy to F_max_ (Fig. 7B,C; *P*=0.6233 versus 0.8405, respectively).

## DISCUSSION

A mouse model of osteocalcin deficiency supports that osteocalcin is a negative regulator of bone formation (Ducy et al., 1996); moreover, the mouse model also implicated osteocalcin as a mediator of skeletal modulation of metabolism, male fertility and cognition (Lee et al., 2007; Oury et al., 2011, 2013). The role of osteocalcin in humans and in human disease remains undetermined (Booth et al., 2013, 2014; Li et al., 2016). Owing to considerable divergence between mouse and human osteocalcin at both the genomic and protein level, the validity of extrapolating results from the osteocalcin-deficient mouse to human disease has been challenged (Booth et al., 2013, 2014). Similarity and synteny between the rat and human osteocalcin gene locus suggest that the rat might be a more appropriate animal model system to investigate osteocalcin function in humans. Here, we report the generation and initial characterization of an osteocalcin-null mutant rat model created using the CRISPR/Cas9 system. As demonstrated in the osteocalcin-deficient mouse model, complete loss of osteocalcin in the rat affected bone structure and function, with increased trabecular bone and increased bone strength. In contrast to the mouse model, our data did not demonstrate increased density of cortical bone based on µCT. Furthermore, the osteocalcin-null rat model did not develop obesity, insulin resistance or glucose intolerance. However, the data reported here are limited to adult 5-month-old animals, and further studies are underway at additional time points in males and females to determine whether additional phenotypes will be detected based on age and/or sex.

Given that the null mutant rat model did not display a striking metabolic phenotype as seen in the osteocalcin-deficient mouse model, the data reported here support that results generated using the mouse model might not be translatable to the role of osteocalcin in humans. The major genomic differences between mice and humans include that: (1) osteocalcin is a highly conserved gene; however, the human and mouse genes share only ∼68% identity versus the human and rat genes sharing ∼75% identity in the osteocalcin coding sequence; (2) the mouse osteocalcin gene locus underwent a triplication event resulting in two functional copies of osteocalcin being expressed in bone and an additional copy expressed in non-osteoid tissues (Desbois et al., 1994); both the human and rat osteocalcin gene loci contain a single copy; and (3) vitamin D downregulates expression of the mouse osteocalcin genes, whereas it upregulates expression of the human and rat gene (Kesterson et al., 1993; Arbour et al., 1995; Javed et al., 1999; Booth et al., 2013). There are also significant differences between the osteocalcin-deficient mouse model and the null mutant rat model we developed. First, the mouse model presumably still has a functional copy of *Bglap-3*, whereas the rat model has a complete loss of osteocalcin protein. Second, the mouse model included deletion of two putative *Paqr6* genes, whereas the rat model represents a selective knockout of osteocalcin only. Although the two deleted *Paqr6* genes are likely to be pseudogenes, it is intriguing to consider that deletion of a progestin and adiponectin receptor family member might be predicted to display metabolic, gonadal and cognitive alterations (Michalakis and Segars, 2010; Patel et al., 2012; Pang et al., 2013; Singh et al., 2013). Although off-target effects are described using CRISPR/Cas9 methodology (Wang et al., 2015), the use of an outbred strain with several different homozygous null mutant and compound heterozygous lines that all resulted in a complete loss of osteocalcin protein provides strong evidence that the observed phenotypes are due solely to loss of osteocalcin gene function.

Given that osteocalcin is the most abundant noncollagenous protein in bone, it might be surprising that complete loss of the protein does not result in a more pronounced skeletal phenotype. However, it will also be important to investigate the phenotype in the axial skeleton and at additional time points. In addition, given that osteocalcin acts primarily as an extracellular bone matrix protein, this might indicate functional redundancy with other bone matrix proteins. Regardless, both the previously published mouse model of osteocalcin deficiency and the rat model of complete loss of osteocalcin described here display an increase in bone strength, supporting the idea that osteocalcin functions as a negative regulator of the skeleton. Understanding of the molecular mechanisms involved in promoting bone density has been important to the development of multiple drug therapies for osteoporosis, such as the capthepsin K inhibitors Odanacatib (Eisman et al., 2011) and Balicatib (Adami et al., 2006), anti-SOST antibodies (Padhi et al., 2011), anti-Dkk1 antibodies (Hoeppner et al., 2009), and GSK3β and Sfrp1 inhibitors (Kulkarni et al., 2006; Moore et al., 2009). Bisphosphonates, a standard treatment used in osteoporosis, function to decrease osteoclast-mediated bone resorption (Tsubaki et al., 2014). Previous *in vitro* studies have suggested that osteocalcin might recruit osteoclast precursors and increase subsequent differentiation of these cells into osteoclasts (Malone et al., 1982; Mundy and Poser, 1983; Chenu et al., 1994), suggesting that osteocalcin might be a novel target for treating osteopenia or osteoporosis.

Moreover, previously published data have suggested that osteocalcin might have relevance to bone disease. For example, loss of *Twist* expression in the mouse inhibited osteocalcin expression and animals displayed the premature cranial ossification of Saethre–Chotzen syndrome (Yousfi et al., 2001). Second, the osteocalcin promoter contains a cis-acting element termed *ose2*, a binding site for the osteoblast transcriptional regulator *Runx2*, which has been implicated in cleidocranial dysplasia in humans and mice (Mundlos et al., 1995; Otto et al., 1997). Finally, a polymorphic marker, D1S3737, is tightly linked to the human osteocalcin gene. One allele of D1S3737 is significantly associated with bone mineral density in postmenopausal women when compared with controls, suggesting that genetic variation at the osteocalcin gene locus might predispose some women to osteoporosis (Raymond et al., 1999). Increased bone density, then, might be protective from osteoporosis and fracture prevalence; however, too much of an imbalance can result in skeletal dysplasia, joint replacements, mandible enlargement, bone pain and nerve compression. Thus, osteocalcin might represent a target for the development of novel therapeutic agents for diseases of bone dysmetabolism. The osteocalcin-null rat model we have developed here might be pivotal to elucidating the molecular mechanisms by which osteocalcin acts on bone remodeling. To this end, future studies will focus on primary osteoblast and osteoclast culture models to assess the consequences of osteocalcin deletion in the rat.

In conclusion, we report that complete loss of osteocalcin in rats results in increases in trabecular bone volume and density and bone strength. To our knowledge, this is the first genetically targeted allele in rats to produce a bone phenotype. The complete loss of osteocalcin did not affect body weight or composition. In contrast to results in the mouse model of osteocalcin deficiency, insulin sensitivity might be increased in the rat model. Given the limitations of the mouse model, the rat might be a more appropriate animal model system to investigate osteocalcin function, particularly as it pertains to relevance in human disease.

## MATERIALS AND METHODS

### Use of animals

All rats were obtained from Taconic Farms, Inc. (Hudson, NY). Phenotyping assays were conducted in homozygous null and compound heterozygous animals of multiple combinations of confirmed null alleles. The single copy number of the *Bglap* gene was demonstrated in the presence of only two bands in HMAs of heterozygous animals and of only one band in the homozygous wild-type and null mutant animals. Additionally, following genotyping, all null mutant animals had total loss of protein by serum ELISA, western blot and immunohistochemistry (Fig. 2D–F). All procedures using the rat (*Rattus norvegicus*) in this project were conducted with the approval of the IACUC and the UAB Animal Resources Program (ARP), with only the requested number of animals needed for completion of the project. The ARP has been accredited by AAALAC since 1971. UAB is registered as a research institution with the USDA and is in full compliance with the NIH policy on animal welfare as filed with the Office for Protection from Research Risks on April 16, 1979, and reaffirmed on April 1, 1990 (#A3255-01).

### CRISPR sgRNA design and synthesis

CRISPR guide RNAs were designed using the MIT Server to target exons 1 and 2 in the rat osteocalcin locus. Rbglap-CRISPR1 (Exon 1, reverse strand) was 5′-CAGAGAGGCAGAATGCAGTC*AGG*-3′; Rbglap-CRISPR2 (Exon 2, reverse strand) was 5′-TTTGTCAGACTCAGAGTCGC*TGG*-3′ [nucleotides in italics represent the protospacer adjacent motif (PAM), the sequence required for CRISPR/Cas9 targeting]. Annealed oligonucleotides encoding the guideRNA were cloned into a plasmid vector (Hwang et al., 2013) and confirmed by sequencing. Single guide RNAs (sgRNAs) were generated using the Ampliscribe T7 RNA transcription kit (Epicenter, Madison, WI) and purified. Cas9 mRNA was *in vitro* transcribed using the pCS2-nCas9n plasmid (gift of Wenbiao Chen, Department of Molecular Physiology and Biophysics, Vanderbilt University, USA; Jao et al., 2013) and SP6 *in vitro* transcription kit (CellScript Inc., Madison, WI). The final concentration of Cas9 mRNA and CRISPR sgRNA in the injection solution were 25 ng µl^−1^ and 50 ng µl^−1^, respectively.

### Gonadotropins

Female Sprague-Dawley rat embryo donors from 3 weeks of age to adulthood were administered 20 IU of PMSG (Sigma, St Louis, MO) at 3 days prior to the day taken as conception followed by 30 IU of HCG (Sigma, St Louis, MO) 2 days later to induce superovulation. At 5 days prior to the day taken as conception recipient Sprague-Dawley female rats over 8 weeks of age were administered 40 IU of LHRHa (Sigma, St Louis, MO) to synchronize estrous cycles. Donor and recipient females were mated to stud and vasectomized Sprague Dawley males, respectively, on one day prior to the day taken as conception.

### Collection of embryos

At day 0.5 post-conception, the synchronized donor female was humanely sacrificed using CO_2_ followed by cervical dislocation. The animal was placed in dorsal recumbency and the abdomen was liberally scrubbed with betadine. The abdomen was carefully opened to expose the abdominal cavity and the uterine horns were sequentially grasped carefully with blunt forceps to allow tracing to the corresponding ovary. The oviduct was isolated, excised, and flushed with sterile medium to expose the cumulus masses containing fertilized embryos. Embryos were cultured in KSOM (Millipore, Darmstadt, Germany) prior to microinjection.

### Microinjection

Fertilized embryos were placed in M2 medium (Millipore, Darmstadt, Germany) and covered in embryo-tested mineral oil on a Leitz/Leica Laborlux S Nomarski DIC microscope (Leica, Wetzlar, Germany). The CRISPR/Cas9 solution was injected directly into the pronuclei using an injection needle and holding pipette controlled by micromanipulators.

### Embryo transfer

Instruments were sterilized by bead sterilization for 5 s. Anesthesia was induced in the recipient rat by placement in a chamber with 1.0–1.5 l min^−1^ of 5% isoflurane with oxygen as a carrier gas and maintained during surgery with 3% isoflurane by placement of the nose of the pre-anesthetized rat in a nose cone after subcutaneous injection of 0.10 ml of 0.3 mg ml^−1^ buprenorphine (Reckitt Benckiser Pharmaceuticals, Inc., Richmond, VA) and 0.2 ml of 5 mg ml^−1^carprofen (Zoetis, Florham Park, NJ). The lower back of the recipient rat was shaved above the left uterine horn, and placed on a sterile tissue on the stage of the microscope. The oviduct was exposed through an incision in the abdominal wall. The manipulated embryos were then injected into the oviduct by gentle pressure through the pipette into the ostium of the oviduct. The reproductive tract was then carefully replaced in the abdomen, the abdomen was closed using suture in the body wall, and the skin was closed with a single wound clip. Post-surgical analgesics were provided [subcutaneous injection 0.2 ml carprofen (5 mg ml^−1^) after 24 h].

### Animal identification

Animals were identified by cage card, sex and through unique ‘ear tags’ consecutively numbered that were affixed at weaning.

### Biopsies

Tail biopsies were performed when animals were weaned. A 5–7 mm portion of the distal segment of the tail was cut and the remainder cauterized for analysis. Genomic DNA was purified from the lysed tail samples.

### Identification of founders

Founder animals were identified by PCR using primers flanking the target loci that amplified a 601-bp fragment in wild-type animals (Rnbglap-genF1, 5′-GGCTCAGGCAGTGGATATAAA-3′; Rnbglap-genR1, 5′-CACAACTCCTCCCTACCAATATG-3′). Positive samples were confirmed by modified Sanger sequencing. Heteroduplex formation of amplified fragments was facilitated by denaturing the PCR samples at 95°C for 10 min and slowly cooling the samples to 4°C over ∼20 min to enable renaturation. The re-annealed samples, which included homoduplexes and heteroduplexes, were run on 6% polyacrylamide-TBE gels at 100 V for 45 min before staining with ethidium bromide to visualize bands under UV light. Samples showing heteroduplex mobility shifts were cloned into a plasmid vector (pCR 2.1) using the TOPO-TA kit (Thermo Scientific, Waltham, MA). Recombinant plasmids with inserts were isolated and subjected to Sanger sequencing to obtain sequence information of modified alleles.

### Genotyping procedure

The following PCR primer sets were used to identify the indel alleles found in F0, F1 and F2 animals: Rnbglap-genF1, 5′-GGCTCAGGCAGTGGATATAAA-3′ and Rnbglap-genR1, 5′-CACAACTCCTCCCTACCAATATG-3′ (601 bp); Rnbglap-genF2, 5′-AAGTCCCACACAGCAACTC-3′ and Rnbglap-genR2, 5′-CGGAGTCTATTCACCACCTTAC-3′ (474 bp); and Rnbglap-genR3, 5′-CTCTCTGGTAGTTTGTCCCTTC-3′ and Rnbglap-genF3, 5′-CACAGCATCCTTTGGGTTTG-3 (329 bp).

### Western blotting

Tissue samples were homogenized in ice-cold T-PER lysis buffer (Thermo Scientific, Waltham, MA) with a protease inhibitor tablet (Complete Mini, EDTA-free; Roche Diagnostics, Mannheim, Germany). Protein concentration was determined using the BCA assay (Thermo Scientific, Waltham, MA). For immunoblotting, protein was loaded on 15% SDS-PAGE gels and separated by electrophoresis, transferred to Immobilon-P^SQ^ membrane (Millipore, Darmstadt, Germany) at 100 V for 50 min, and crosslinked using 2.5% glutaraldehyde for 1 h at room temperature. Membranes were blocked with 5% nonfat dry milk for 1 h at room temperature, then incubated overnight with a monoclonal anti-osteocalcin antibody [Abcam, Cambridge, MA, cat. no. ab13420, validated in rat in Esteves, et al. (2013)] diluted 1:2500 at 4°C, followed by incubation with horseradish-peroxidase-conjugated goat anti-mouse-IgG secondary antibody. The immunoblots were visualized by chemiluminescence (Thermo Scientific, Waltham, MA).

### Immunohistochemistry

Immunohistochemical analyses of osteocalcin was performed as previously described (Cook et al., 2013). Briefly, excised femurs were submerged in 10% neutral buffered formalin (Sigma, St Louis, MO) for fixation before decalcification with 10% EDTA and paraffin embedment. 5-μm-thick sections of formalin-fixed paraffin-embedded decalcified femoral tissue sections were deparaffinized in xylene and rehydrated in graded alcohols. For antigen retrieval, slides were immersed and boiled for 20 min in a diluted (1:30), pH 9.0 antigen unmasking solution (Vector Laboratories, Burlingame, CA). Slides were incubated in a horse serum blocking solution (ImmPRESS system, Vector Laboratories) for 1 h followed by incubation with monoclonal anti-osteocalcin antibody [1:200; Abcam, Cambridge, MA, cat. no. ab13420, validated in rat in Esteves et al. (2013)] in phosphate-buffered saline solution containing 1% bovine serum albumin. Appropriate secondary antibody (ImmPRESS, Vector Laboratories) was applied and slides were incubated in DAB (3,3′-diaminobenzidine) peroxidase substrate solution (Dako). Each slide was then incubated with Harris hematoxylin counterstain (Fisher Scientific, Waltham, MA). Cells positive for osteocalcin stained brown.

### DXA

*In vivo* body composition of the rats was assessed using the GE Lunar Prodigy DXA with Small Animal Software (GE, Madison, WI; v.6.10) as previously described and validated (Moreau et al., 2001; Bertin et al., 1998). The rats were anesthetized with a constant flow of 4% isoflurane in oxygen. They were then placed in a prostrated position on the DXA and scanned using the Small Animal Software (v.6.10). Each scan took ∼5 min and the resulting data were analyzed by drawing a region of interest that included the entire rat. Data obtained from this scan included total body fat mass, lean mass, BMD and BMC.

### Insulin tolerance test and glucose tolerance test

Rats were fasted for 4 h prior to the ITT conducted in the afternoon and overnight prior to the GTT conducted in the morning. After the fast, the rats were weighed and placed in a clear restraint tube which allowed easy access to the tail for blood sampling. The tip of the tail (1 mm) was cut with a scalpel for the baseline sample. Blood was collected from this same point throughout the test, without needing to cut the tail again. Approximately 2 µl of blood per read was analyzed for glucose content using the Alphatrak analyzer and strips (Zoetis, Florham Park, NJ). Insulin or glucose was injected into the peritoneal cavity after the baseline glucose measurement and blood glucose was measured at the following time points post injection: 15, 30, 45, 60, 75, 90, 105 and 120 min. Doses of insulin and glucose were dependent on the glucose status of the animals and were 0.75 IU kg^−1^ and 1.50 mg g^−1^, respectively.

### µCT

Wild-type littermates and osteocalcin-null rats of 5 months of age were sacrificed and their femurs were dissected. Excised rat femurs were scanned using the Scanco µCT40 desktop cone-beam µCT scanner (Scanco Medical AG, Brüttisellen, Switzerland). The femur was placed inverted in a 20-mm diameter scanning holder and scanned at the following settings: 20-mm resolution, 70 kVp and 114 µA with an integration time of 200 ms. Scans were automatically reconstructed into 2D slices and all slices were analyzed using the µCT Evaluation Program (v.6.5-2, Scanco Medical, Brüttisellen, Switzerland). For the cortical analysis, the bone was scanned at the midshaft of the bone for a scan of 50 slices. The region of interest (ROI) was drawn on every slice and fitted to the outside of the cortical bone, to include all the bone and marrow. The threshold for cortical bone was set at 316 (grayscale value). The 3D reconstruction was performed using all of the outlined slices. Data were obtained on bone volume (BV), total volume (TV), BV/TV, bone density and cortical thickness. An additional analysis was performed using the software to measure the periosteal circumference using the same outlines. For the analysis of endosteal circumferences, a new ROI was drawn and fitted to the inside of the cortical bone. The same analysis was then performed as for the periosteal circumference. For the trabecular bone, the scan was started distal to the growth plate and consisted of 312 slices. The region of interest started at the point on the scan where the condyles ended. From this point, 200 slices were outlined on the inside of the cortical bone, enclosing only the trabecular bone and marrow. Trabecular bone was thresholded at 211 (grayscale value) and the 3D analysis performed on the 200 slices. Data were obtained on trabecular bone volume, total volume, thickness, density, separation and number.

### Biomechanical strength testing

Three-point bending tests were completed using an MTS 858 MiniBionix (MTS Systems Co., Eden Prairie, MN, USA) equipped with a 15,000 N load cell (calibrated to 1500 N). Upon killing, the femurs were harvested, cleaned of soft tissues, wrapped in saline-moistened gauze and frozen until use. The bones were placed onto a custom three-point bending apparatus such that primary loading occurs in the posterior to anterior direction. The rate of deformation was established (typically 0.1–0.5 mm s^−1^), and data recorded with a sampling rate of 100 Hz using MTS Basic Testware (MTS Systems Co., Eden Prairie, MN) with parameters being time, axial force and deflection. These data were imported into Excel for analysis and the maximum force, maximum bending moment, stiffness (the slope of the force-displacement curve), and energy to maximum force (area under the force–displacement curve from the point of contact to the point of maximum force) were calculated.

### Collection of serum

Whole blood was collected into 1.5 ml tubes at the time of killing by cardiac puncture with a 26 gauge needle. Blood was allowed to clot at room temperature for 30 min before centrifugation at 1000 ***g*** for 15 min. Serum was collected into 1.5 ml tubes and stored at −20°C.

### Enzyme-linked immunosorbent assay

A sandwich-type EIA Rat Osteocalcin High Sensitive EIA Kit (Takara Bio Inc., Nojihigashi, Japan; cat. no. MK147) was used according to manufacturer instructions to measure osteocalcin in serum from osteocalcin-null and wild-type male rats.

### Statistical analyses

Outcomes were analyzed using GraphPad Prism software (La Jolla, CA) by two-tailed parametric unpaired *t*-tests with a 95% confidence level or a s.e.m., or as the area under the curve, with one-way ANOVA, two-way ANOVA or ANCOVA as appropriate. *P*≤0.05 was considered significant. Outliers were determined by Grubb's outlier test (a=0.05) and excluded from analyses.
